# The Research and Application of Visual Saliency and Adaptive Support Vector Machine in Target Tracking Field

**DOI:** 10.1155/2013/925341

**Published:** 2013-12-02

**Authors:** Yuantao Chen, Weihong Xu, Fangjun Kuang, Shangbing Gao

**Affiliations:** ^1^School of Computer Science and Engineering, Nanjing University of Science & Technology, Nanjing 210094, China; ^2^School of Computer and Communication Engineering, Changsha University of Science & Technology, Changsha 410114, China; ^3^Department of Computer Science and Technology, Hunan Vocational Institute of Safety & Technology, Changsha 410151, China

## Abstract

The efficient target tracking algorithm researches have become current research focus of intelligent robots. The main problems of target tracking process in mobile robot face environmental uncertainty. They are very difficult to estimate the target states, illumination change, target shape changes, complex backgrounds, and other factors and all affect the occlusion in tracking robustness. To further improve the target tracking's accuracy and reliability, we present a novel target tracking algorithm to use visual saliency and adaptive support vector machine (ASVM). Furthermore, the paper's algorithm has been based on the mixture saliency of image features. These features include color, brightness, and sport feature. The execution process used visual saliency features and those common characteristics have been expressed as the target's saliency. Numerous experiments demonstrate the effectiveness and timeliness of the proposed target tracking algorithm in video sequences where the target objects undergo large changes in pose, scale, and illumination.

## 1. Introduction

Target tracking has attracted a lot of attention in computer vision due to its fundamental importance for many vision applications such as visual surveillance, traffic safety monitoring, and abnormal activity detection. And many successful techniques of target tracking have been proposed in the last several decades [1]. The applicability of the techniques in general scenarios, however, is still very limited due to practical difficulties: appearance variations (e.g., illumination, viewpoint, and background changes), occlusions, complex backgrounds, and so forth. These difficulties are inevitable in practical applications and thus noticeably aggravate this problem [2]. To overcome these problems, many researchers have proposed numerous target tracking methods.

In many traditional approaches, various kinds of low-level observation models have been used for object tracking, such as feature points [3], lines or templates [4], moving areas [5], and color appearance models [6]. The common framework for target tracking algorithm mainly includes mean-shift method using Kalman filter and particle filtering algorithm. The particle filter is a filtering algorithm based on Bayesian inference, through nonparametric sequential Monte Carlo methods. The particle filter is not linear and Gaussian distribution systems to meet the restrictions are widely used in navigation, machine vision, target tracking and so on. We can use the variety of particle filtering features, colors, and edge contour. They are more commonly used as two representations of target features. Wherein the color feature is not sensitive to noise and partial occlusion, but sensitive to changes in illumination, and when the target's color and the background's color distribution are similar to the distribution, it cannot correctly distinguish the object and the background. Edge contour feature robust to illumination changes, but in the case of complex backgrounds, higher computational complexity, cannot guarantee the real-time state.

The use of visual attention mechanism of the visual information on the input selection process, visual information from the mass filter has found out a small amount of useful information to the visual tracking algorithm [7]. The information processing can reduce the amount of computation to improve the efficiency of information processing. Therefore, some researchers have gradually begun to study visual saliency attention based on object tracking method. Some researchers target tracking problem as the human visual attention transfer process to establish a computational model of visual attention. The focus of visual attention has shifted through sight target detection and target tracking [8, 9], but the goal is not always to be tracked the most significant and therefore full advantage of significant information and track deviation results. However, [10] has considered only static significance, ignoring the dynamic characteristics significantly, while [11] only for the case of stationary background target tracking problem is studied.

We have proposed a novel combination of adaptive support vector machine (ASVM) based on support vector machine (SVM) and visual saliency feature extraction algorithm for the moving target tracking. The new algorithm has utilized ASVM as the tracking algorithm's framework, and the use of visual saliency has measured the model to calculate the target saliency features. They have included many common characteristics as the target for model representation in order to overcome the use of the single color feature which brings tracking instability problems. Numerous experiments demonstrate an effective solution because the target deformation, illumination change, the target's background color distribution similar difficulties arising track to achieve the robust target tracking algorithm.

## 2. Adaptive Support Vector Machine

### 2.1. Sample Selection Algorithm

During the image preprocessing and feature extraction, we firstly need to mark the given sample selection of specific definition.

**Definition 1**

For each training sample pixel of (*x*_*i*_, *y*_*i*_), *l*(*x*_*i*_) is called the sample (*x*_*i*_, *y*_*i*_) of the sample pixel tags. The assignment conditions are shown as follows:

(1) $$\begin{array}{c} l\left(x_{i}\right)=\{\begin{array}{cc} 1, & \left(x_{i},y_{i}\text{ isn't support vector}\right),\\ 0, & \left(x_{i},y_{i}\text{ is support vector}\right). \end{array} \end{array}$$

The definition in the role of the labeled sample is used to determine whether *r*(*x*_*i*_) discarded mainly based on SVM of incremental learning. It can be used to store a number of occurrences of *l*(*x*_*i*_) = 1. When *l*(*x*_*i*_) = 1, *r*(*x*_*i*_) had been based on the data in the original plus one operation. When *r*(*x*_*i*_) had reach specific thresholds, you can sample the pixel from the entire training set (*x*_*i*_, *y*_*i*_).

Deepening of the training process execution can result in some samples happening “oscillation sample”. In order to solve the problem, the threshold should be introduced to represent the training set for the sample point data as nonsupport vector frequency maximum threshold values need to consider the training time and training precision balance factor. When *r*(*x*_*i*_) ≥ *l*, the pixel sample expressed by (*x*_*i*_, *y*_*i*_) can be eliminated from the training set. The algorithm can reduce the “hunting” phenomenon of the sample pair classifiers related impact in preprocessing and feature extraction.

### 2.2. Increment and Decrement of ASVM Learning Algorithm

Since many of the existing incremental learning algorithms and lack of learning algorithms for reducing training set data selectively eliminated, so that the processing time will largely affect the accuracy of SVM. Most of the original incremental learning algorithms are executed in an incremental process which discards pixel samples from nonsupport vectors, but with subsequent incremental ongoing training process, before being discarded, nonsupport vectors are likely to become the support vector. Moreover, it is likely to effectively deal with some important information in a single training sample, and it directly discards the pixel with nonsupport vectors. It is results in decreased accuracy of the classification process. To solve the above problem, Section 2.1 had made the defined markers to mark a sample of the treatment. The effect can be achieved from increment and decrement ASVM learning algorithm.

The increment and decrement learning algorithm's idea is shown as in the following description. Firstly, the introduction of threshold *l* can be expressed in the training set for tolerating a non-pixel sample (*x*_*i*_, *y*_*i*_) maximum number of support vectors. The sample pixel (*x*_*i*_, *y*_*i*_) into the training set samples and the sample of the recording pixel (*x*_*i*_, *y*_*i*_) non-specific number of support vectors *n*, when the number *n* has reached a predetermined threshold value, will be pixels of a sample after *l*(*x*_*i*_, *y*_*i*_) drop operation performed from the training set. The following steps have described the specific description of increment and decrement ASVM learning algorithm.

**Algorithm 2**

Consider the increment and decrement adaptive support vector machine learning algorithm.

**Step 1 (initialization process)**

For the specific setting of the threshold value of *l*, get a sample point until SVM support vector types and non-pixel samples appear and set the current pixel to obtain the sample set to *T*. *T* is the training set to perform the training process, thereby obtaining a sample labeled *l*(*x*_*i*_), so that *r*(*x*_*i*_) = *l*(*x*_*i*_), *i* = 1,…, |*T*|.

**Step 2**

The current pixel sample set is *T*, and |*T*| = *m*. Newly acquired pixel sample had been expressed as (*x*_*m*+1_, *y*_*m*+1_), so that *r*(*x*_*m*+1_) = 0. Put *T* = *T* ∪ {(*x*_*m*+1_, *y*_*m*+1_)} as the training set performs a retraining process.

**Step 3**

Consider

 (3.1) *T*_*d*_ ← *ϕ*
 (3.2) for *j* = 1 to *m*
 (3.2.1) if *l*(*x*_*j*_) = 1 (*x*_*j*_ is not support vector), then *r*(*x*_*j*_) ← *r*(*x*_*j*_) + 1. If *l*(*x*_*j*_) ≠ 1, *r*(*x*_*j*_) remains;
 (3.2.2) if *r*(*x*_*j*_) ≥ *l*, then *T*_*d*_ ← *T*_*d*_ ∪ {*x*_*j*_, *y*_*j*_};
 (3.3) if *T*_*d*_ ← *ϕ*, then *T* = *T* − *T*_*d*_ to become the training set to perform decrement process of learning.

**Step 4**

When there is a new sample, then transfer to Step 2 to continue to perform incremental learning (3.3) and decrement process (3.2).

Algorithm 2 had described the adaptive learning algorithm using increment and decrement ASVM learning method. The existing increment learning algorithms and decrement learning algorithms have differences in the third step of Algorithm 2. Before discarding sample pixel (*x*_*i*_, *y*_*i*_), Algorithm 2 uses (*x*_*i*_, *y*_*i*_) to depict the determining execution whether the number of support vectors exceed the pre-determined threshold of *l*. If it exceeds the threshold value, then the sample pixel (*x*_*i*_, *y*_*i*_) concentration dropped from the sample. If it does not exceed the threshold value, then the sample (*x*_*i*_, *y*_*i*_) pixels remain. The threshold may be the introduction of adaptive strategies and adaptive strategy aims to improve the training accuracy and reduce training time.

## 3. Visual Saliency Feature Extraction in ASVM Tracking Algorithm

**Figure 1:**
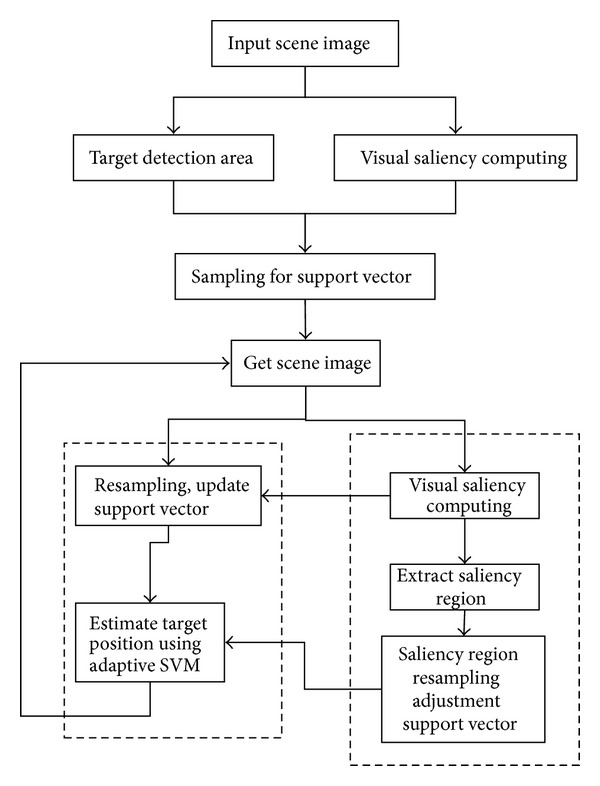
Visual saliency feature extraction algorithm flow diagram using adaptive SVM.

Figure 1 is based on the visual saliency feature extraction and adaptive SVM target tracking algorithm flow diagram.

The object tracking algorithm had been described for the specific process as follows.

 (1) The initialization process: as *t* = 0, the image is acquired for the scene, depending on the scene image for relevant target, setting the initial state of relevant targets: *X*_0_ = (*x*_0_, *y*_0_, *h*_*x*_, *h*_*y*_), (*x*_0_, *y*_0_) coordinates of the center position of the target. (*h*_*x*_, *h*_*y*_) is the width of the target area and the target area height. According to Section 4, the method of calculation of the various parts of the scene image saliency features and similarity values, for the detection of the target area, calculates the target color space histograms *H*_*c*_ and visual saliency feature histogram *H*_*s*_ which has become the target feature representation model. *H*_*c*_ and the color histogram feature visual saliency *H*_*s*_ histogram calculation in Section 4 will be described. The initial distribution for the point {*x*_0_^*i*^}_*i*=1_^*N*^ of *N* pixels is sampled for each pixel and the weight *w*_0_^*i*^ of the importance is assigned to 1/*N*.
 (2) If *t* = *t* + 1, it can continue to get the visual scene images to calculate its significance. The application of autoregressive model is shown in
  (2) $$\begin{array}{c} X_{t}=AX_{t-1}+v_{t-1}. \end{array}$$
 In formula (2), *v*_*t*−1_ is the process noise. *A* is the state transition function.
 We can use adaptive learning algorithm and increment and decrement learning method in the target state transition model for processing and support vector {*x*_*t*_^*i*^}_*i*=1_^*N*^ sample. The support vector {*x*_*t*_^*i*^} had been calculated for each color space histogram *H*_*c*_^*i*^ and visual saliency histogram of *H*_*s*_^*i*^.
 (3) Using each of the supported equation $d^{F}\left(x_{1},x_{2}\right)=\sqrt{K\left(x_{1},x_{1}\right)-2K\left(x_{1},x_{2}\right)+K\left(x_{2},x_{2}\right)}$ to calculate the support vector {*x*_*t*_^*i*^} and the distance between the target areas, the target has support vector similarity metrics.
 (4) According to the visual saliency measure of calculation result, the effective extraction of the image scene significant regions. Use $d^{F}\left(x_{1},x_{2}\right)=\sqrt{K\left(x_{1},x_{1}\right)-2K\left(x_{1},x_{2}\right)+K\left(x_{2},x_{2}\right)}$ to calculate for each region which is significantly related to the distance between the target models. We can select the smallest distance from the salience regions to replace a large number of support vectors. So, they can be called the adjusted support vector.
 (5) For calculating the weight of each support vector and normalizing it, the specific method will be described in Section 4.
 (6) Making Section 2 and decrement learning adaptive incremental learning method is to get the sample optimal target state.
 (7) Based on support vector collection of support vector weights, high weight retention support vector, discarding the low weight of the support vector, updates support vector reaches of *N*. At last, return to Step 2 implementation of the overall process.

## 4. Proposed Approach Description

### 4.1. Color Model and Similarity Measure

Adaptive SVM can be used to treat a variety of relevant characteristics expressed to detect target objects. About the color feature part, due to the color block part of the noise signal and the fact that it is not sensitive, the calculation process is simple. The color characteristics have widely been used. The HSI color space and the human visual system characteristics are similar to their own features. We can use the formula (3) to depict the scene image from the RGB color space. It can be switched to the HSI color space

(3) $$\begin{array}{c} H=\frac{1}{360}\left[90-\arctan\left(\frac{F}{\sqrt{3}}\right)+\{\begin{array}{cc} 0 & G>B\\ 180 & G<B \end{array}\right],\\ S=1-\left[\frac{\min\left(R,G,B\right)}{I}\right],\\ I=\frac{\left(R+G+B\right)}{3},\\ F=\frac{2R-G-B}{G-B}. \end{array}$$

The algorithm in Section 3 can be used for the color histogram as the color model of the target areas. It can be supposed that the whole color space is divided into *m* subregions by calculating the image of the color vector scene. It can enter each subregion pixel number of frequencies to obtain a histogram of *m*. The columns have contained the color space histogram. Considering the sample in the target area pixel position on the color distribution for the related effects can increase kernel function *k*(*r*) to spatial information for the integration, namely,

(4) $$\begin{array}{c} k\left(r\right)=\{\begin{array}{cc} 1-r^{2}, & r<1,\\ 0, & r\geq 1. \end{array} \end{array}$$

In formula (4), *r* is a pixel and is the distance between the midpoints of the target area.

The application of *H*_*c*_ = {*p*_*c*_^*u*^(*x*)}, *u* = 1,2,…, *m* can to be expressed in the center region *x* pixel color distribution model. Formula (5) describes the model

(5) $$\begin{array}{c} p_{c}^{u}=C\sum_{i=1}^{N}k\left(\frac{||x_{i}-x||}{a}\right)\delta \left(b\left(x_{i}\right)-u\right). \end{array}$$

In (5), *x*_*i*_ is a pixel region, *N* is the number of pixels in the central region, and *b*(*x*_*i*_) is the color characteristics of the *x*_*i*_. It can be assigned to the corresponding part of the color histogram. *δ*(·) is the Dirac function. $a=\sqrt{w^{2}+h^{2}}$ is represented as the area size. *C* = 1/∑_*i*_^*N*^ *k*(||*x*_*i*_ − *x*||/*a*) is a normalization factor.

### 4.2. Visual Saliency and Similarity Measurement

Color is very sensitive to illumination changes, when the target had been detected and the background color of the color range interval is closed. Only using color feature representation model as a target feature, the object tracking results are often not easy to achieve the desired state. The proposed algorithm can use visual saliency feature fusion and targets' color to be detected as a representation model.

**Figure 2:**
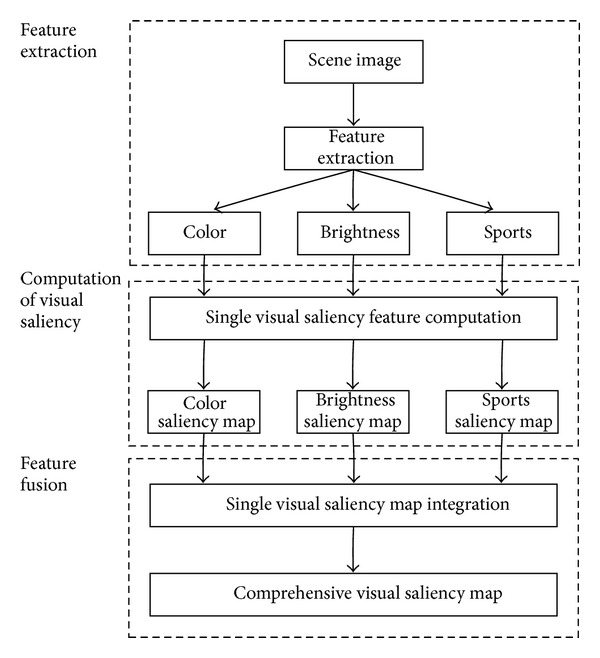
Visual saliency calculation method.

In some small part of the scene image, most of the contents are more than the other to win the human observer visual saliency. The people called these small parts with high visual saliency. Visual saliency measurement by the color characteristics of the scene image, brightness feature, and sports feature together produces an effect, compared with the simple color features. Visual saliency with high robustness, high robustness, and high noise immunity and the visual saliency calculation of specific overall process are shown in Figure 2. 

*(1) Feature Extraction.* The application of (3) in the scene image from the RGB space to HSI space has been switched. The channel of *H*, the channel of *S*, and the channel of *I* as the luminance characteristics are as the color features. The motion characteristics expression is shown in

(6) $$\begin{array}{c} M\left(x,y\right)=f\left(x,y,t\right)-f\left(x,y,t-1\right). \end{array}$$

In (6), *f*(*x*, *y*, *t*) represents pixel's values of (*x*, *y*) at *t* time. *f*(*x*, *y*, *t* − 1) denotes a pixel point (*x*, *y*) at time *t* − 1 which is determined. 

*(2) Visual Saliency Calculation.* Visual saliency feature is the scene image in each area and surrounding environment caused by mutations arising from visual saliency. The greater effects of mutations have significantly higher vision. It can be characterized by calculating the various regions of the figure relative to the surrounding environment with the local characteristics which were compared to calculate the saliency value.

Firstly, the image is featured from the spatial domain into the frequency, so that the image can be obtained from the amplitude spectrum |*F*(*u*, *v*)| and *ϕ*(*u*, *v*). The phase spectrum in two feature images has been expressed as

(7) F(u,v)=1M×N∑x=1M∑y=1Nf(x,y)e−j2πux/Me−j2πvy/N=|F(u,v)|ejϕ(u,v),|F(u,v)|=[R2(u,v)+I2(u,v)]1/2,ϕ(u,v)=arctan(I(u,v)R(u,v)).

In (7), *f*(*x*, *y*) is the pixel (*x*, *y*) of the specific characteristic value. *M* × *N* is the image size.

Image phase spectrum amplitude of features and characteristics of each image have contained a variety of specific information in the image. The characters in the amplitude spectrum of the image information in each of the frequency change and phase information changing spectral characteristics indicate position information. Calculating visual saliency was aimed at each image pixel to measure the significance, looking significantly for larger pixel location. Using the image phase spectrum tectonic features had restored image; the output value has greater saliency location of the pixels in the original image corresponding to eigenvalues of larger changes position, and these positions are in “visual saliency” area. Therefore, only the spectral characteristics of the phase image for the original structure to conduct inverse image using Fourier transform can be restored after reflecting on the various parts of the image was significantly related to the saliency map. It is named as

(8) $$\begin{array}{c} S\left(x,y\right)=\frac{1}{M\times N}\sum_{u=1}^{M}\sum_{v=1}^{N}\phi \left(u,v\right)e^{-j2\pi ux/M}e^{-j2\pi vy/N}. \end{array}$$

*(3) Feature Fusion.* According to the above method of color, brightness, and sports feature, each saliency map can significantly show in these visual features fusion diagram. It is to obtain the final visual saliency figure, namely, as

(9) $$\begin{array}{c} S=w_{c}S_{c}+w_{I}S_{I}+w_{M}S_{M}. \end{array}$$

In (9), *S*_*c*_, *S*_*I*_, *S*_*M*_, respectively, using formula (8) stand for the color characteristics, luminance characteristics, sports characteristics. Visual saliency measurement had been calculated finally to obtain three relevant characteristic time of the visual saliency map. *w*_*c*_, *w*_*I*_, *w*_*M*_ weights represent color weights, brightness weights, and sports weights. It depicts 1/3 concrete representation of the three characteristics significantly averaging feature fusion graph execution.

Integrated visual saliency map is exactly the same size with the scene grayscale image. Each pixel's value represents the corresponding position in the scene image pixel of visual saliency values. The application and feature extraction have part of the same way, and we can get visual saliency distribution model *H*_*s*_. Each saliency model of support vector and the target model were significantly similar between the values and visual saliency map.

*(4) The Right to Calculate the Pixel Value.* The color of each pixel based on models and visual saliency model and target features to represent the value of the degree of similarity between the models used to calculate the weight of each pixel has a value. It is shown as

(10) wti=p(zt ∣ xti)∝(1−αt)e−λ(ρci)2+αte−λ(ρsi)2,αt=ρ−cρ−c+ρ−s.

In (10), *ρ*_*c*_^*i*^ represents *i* pixel of color model and the target color values of the similarity degree between these models. *ρ*_*s*_^*i*^ represent *i* pixel point visual saliency model and the target visual saliency value of the similarity degree between these models. ${\overset{-}{\rho }}_{c}$ is all averages of *ρ*_*c*_^*i*^ and ${\overset{-}{\rho }}_{s}$ is all averages of *ρ*_*s*_^*i*^.

## 5. The Experimental Results and Analysis

### 5.1. Adaptive SVM Numerical Experiments

In order to evaluate the proposed algorithm in the specific performance, the correct rate of the paper from the training, testing accuracy, and CPU execution time of three elements had been compared with ASVM and online incremental learning algorithms in [12]. Using online algorithms directly below the expression, [12] had proposed the online incremental learning algorithm, using ASVM for the proposed adaptive increment and decrement the SVM learning algorithm. The following experiments have been from the linear case and the respective status of implementation of the relevant nonlinear numerical experiments as well.

In the adaptive SVM numerous experiments, firstly select the UCI machine learning database [13] concerning the data sets related numerical experiments. The sample through the training set individually is added to online simulation. Taken *λ* = 1.9/*C*, *C* for the penalty parameter, *ε* is required to achieve 10^−5^. Penalty parameter *C* is selected from the training set through the adjustment set in the training process selecting the optimal value. Numerous experiments on the selected threshold value *l* are achieved by the variety of UCI machine learning databases through constant adjustment and test selected. According to the numerous experiments relevant results, finally finding the threshold value *l* = 4 is to ensure the training success rate, test success rate, and CPU execution time of the optimal solution.

The experimental results in the linear case are shown in Table 1. ASVM on the classification accuracy than online algorithm, the CPU execution time significantly better than the online algorithm, such as for higher dimensional data sets Pima-diabetes. ASVM's execution time is 1.85 seconds, and the online execution time is 15.4012 seconds.

For the nonlinear case, we use the RBF kernel function *K*(*x*, *y*) = exp⁡(−*p*||*x*−*y*||^2^). The nonlinear case numerous results are shown in Table 2. *p* is the parameter of kernel function. According to Table 2, the numerous results to be seen are shown: (1) ASVM executed by the CPU than the online time is significantly smaller; (2) ASVM correct rate training and which testing accuracy is more than the online algorithm is significantly higher.

### 5.2. Calculating Visual Saliency Map Experiment

**Figure 3:**
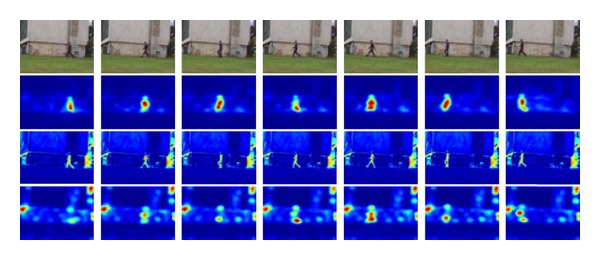
Three cases of visual saliency.

**Figure 4:**
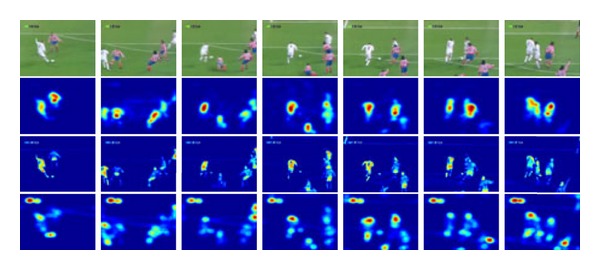
Three cases of visual saliency.

In Figures 3 and 4, the first line of the original video image of each frame image. The second line of image is obtained by considering only the color characteristics of the visual saliency map [14]. The third line of image is obtained by the algorithm and applied ASVM visual saliency map. The fourth line of the image is applied only notable characteristics obtained saliency map [15].

According to Figures 3 and 4, the visual saliency map has comparison results. The application of ASVM algorithm proceeds visual saliency map best reflecting the original image characteristic graphs of the results. The effect is good visual saliency map in the subsequent tracking process which can also play a very good supporting role.

### 5.3. The Testing Experiments of Object Tracking Algorithm

In order to verify the accuracy of the proposed algorithm and effectiveness, the experiment environments have included Intel Pentium 2.6 GHz, 4 G RAM. The simulation testing software has used Matlab 2012b. The focusing algorithm for tracking the effectiveness of tests, including light intensity changes in the target deformation and occlusion of target tracking results, does not introduce significant visual support vector machine algorithm for horizontal contrast.

**Figure 5:**
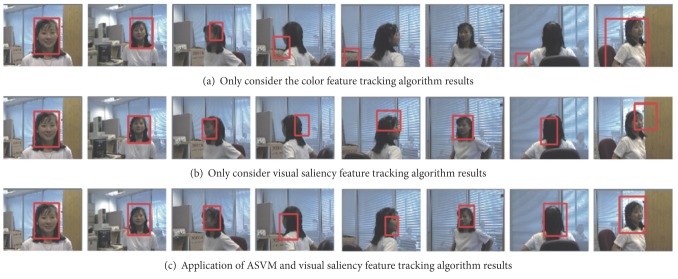
Target rotation, shape, and size variation of the tracking results.

Object tracking experiment is rotated and the changes of shape and size for the video sequence header target tracking as well [16]. The test video sequence has 500 frames of video targets which are moving the camera. The target size, shape, and pose had significantly changed in Figure 5. It demonstrates the detailed tracking results of three cases. The 1, 63, 83, 96, 105, 156, 178, 244 video sequences have respectively the frame [17]. It can be seen in Figure 5 when the target before and after exercise-induced changes in the target size, but not the target color characteristics, does not change significantly. The first two algorithms can be tracked correctly. The turn movements occur when the target and the target color distribution greatly occurred changes, using only a single feature representation model as a target tracking is performed. It will fail and lose the target track. And in addition to the color characteristics of the algorithm, it also joined the target saliency features, with single feature algorithms with high stability and antijamming capability. The algorithm is tested even when the target rotation, shape, and color are changed. It is possible for tracking results to obtain high efficiency.

The algorithm is a novel target tracking algorithm based on the integration visual saliency features and color co-feature model as target detection feature representation model. To ensure the effectiveness of target tracking algorithm, the significant increase in space and time complexity of the algorithm had to ensure the algorithm for real-time results.

## 6. Conclusions

The use of visual saliency features and ASVM is significant in the target tracking algorithm proposed in the paper. The algorithm had been based on the image color feature, brightness feature, and sports feature. Visual saliency measurement execution process, visual saliency features, and color features common characteristics are expressed as the target. In the video sequence for related experiments, the effectiveness and timeliness of object tracking algorithm in the paper have achieved excellent results.

In comparison with the existing target tracking algorithm, the algorithm can avoid the application of single color characteristics caused by target tracking instability. In the larger target attitude change, illumination change, shape change, and the emergence of sheltered cases correctly track the target. Prolonged occlusion and dramatic lighting changes may still cause the tracking algorithm failure. Therefore, in addition to color features and visual salient feature, you can also consider other effective features. Using reasonable means, the model will achieve greater robustness and the goal is to further research efforts for tracking direction.

## Figures and Tables

**Table 1:** Numerous results in the linear case.

Data sets	Size	Algorithm	*C*	Training success rate	Test success rate	CPU execution time
Live disorder	Train: 250∗6	Online	100	98.41%	96.82%	2.4430
	Test: 95∗6	ASVM	20	99.74%	97.14%	1.5079
Letter-recognition	Train: 500∗16	Online	10	98.12%	90.43%	3.6875
	Test: 100∗16	ASVM	10	96.43%	92.14%	1.8594
Pendigits	Train: 700∗16	Online	10	78.18%	75.71%	4.8156
	Test: 500∗16	ASVM	10	81.16%	79.23%	1.4063
Tic-tac-toe	Train: 800∗9	Online	100	76.41%	70.42%	8.8126
	Test: 158∗9	ASVM	10	89.13%	82.45%	1.3468
Pima-diabetes	Train: 800∗21	Online	100	90.14%	86.50%	15.4012
	Test: 200∗21	ASVM	100	92.50%	87.81%	1.8500

**Table 2:** Incremental learning outcomes nonlinear case.

Data sets	Size	Algorithm	(*C*, *p*)	Training success rate	Test success rate	CPU execution time
Iris	Train: 150∗4	Online	100, 0.5	98.00%	92.34%	1.6209
	Test: 50∗4	ASVM	20, 0.025	100.00%	97.74%	1.0013
Live disorder	Train: 220∗6	Online	100, 0.01	97.13%	94.72%	3.0049
	Test: 125∗6	ASVM	100, 0.05	100.00%	96.75%	1.9227
Letter-recognition	Train: 400∗16	Online	10, 0.025	96.40%	88.34%	5.8750
	Test: 200∗16	ASVM	20, 0.025	100.00%	91.50%	2.2406
Waveform	Train: 400∗21	Online	10, 0.025	96.40%	88.34%	5.8750
	Test: 200∗21	ASVM	20, 0.025	100.00%	91.50%	2.2406
Pendigits	Train: 700∗16	Online	10, 0.025	82.12%	70.60%	9.3198
	Test: 300∗16	ASVM	100, 0.05	88.34%	79.60%	2.7969
